# Microstructure and Thermoelectric Characterization of Composite Nanofiber Webs Derived from Polyacrylonitrile and Sodium Cobalt Oxide Precursors

**DOI:** 10.1038/s41598-020-66667-6

**Published:** 2020-06-15

**Authors:** Kyoung Moon Ryu, Young Hun Kang, Song Yun Cho, Sang Hoon Lee, Young Chul Choi, Min Su Kim, Young Gyu Jeong

**Affiliations:** 1 Department of Advanced Organic Materials and Textile System Engineering, Chungnam National University, Daejeon, 34134 Republic of Korea; 2 Division of Advanced Materials, Korea Research Institute of Chemical Technology, Daejeon, 34114 Republic of Korea

**Keywords:** Engineering, Materials science

## Abstract

We report the microstructure and thermoelectric properties of composite nanofiber webs, which were fabricated by dual-electrospinning of polyacrylonitrile (PAN) and sodium cobalt oxide (NaCo_2_O_4_) precursor solutions with different input compositions and following heat-treatment at 600–900 °C for simultaneous carbonation and calcination. The SEM and EDS mapping images revealed that PAN-derived carbon nanofibers (CNFs) and NaCo_2_O_4_-based ceramic nanofibers coexisted in the composite nanofiber webs and that their relative contents could be controlled by the input compositions. The Seebeck coefficient increased from ~26.77 to ~73.28 μV/K and from ~14.83 to ~40.56 μV/K with increasing the relative content of NaCo_2_O_4_ nanofibers in the composite nanofiber webs fabricated at 700 and 800 °C, respectively. On the other hand, the electrical conductivity of the composite nanofiber webs increased with the decrement of the relative content of NaCo_2_O_4_ nanofibers as well as the increment of the heat-treatment temperature. Owing to the opposite contributions of NaCo_2_O_4_ nanofibers and CNFs to the Seebeck coefficient, electrical conductivity and thermal conductivity, a maximum power factor of ~5.79 μW/mK^2^ and a figure of merit of ~0.01 were attained for CNF/NaCo_2_O_4_-based composite nanofiber webs fabricated at 45 wt% input composition of NaCo_2_O_4_ and at heat-treatment of 700 °C_._

## Introduction

Thermoelectric materials and devices have a lot of potential because they have the advantage of using eco-friendly methods to generate electrical energy using the waste heat from the industry and human or the heat in nature^1^. Those materials and devices are based on the thermoelectric effect, which is the reversible conversion between electrical energy and thermal energy. The performance of the thermoelectric materials can be evaluated as a thermoelectric figure of merit, *ZT* = *S*^2^σ*T*/*κ*, *S* is the Seebeck coefficient, σ is the electrical conductivity, *κ* is the thermal conductivity and *T* is the absolute temperature^2–5^. Therefore, high electrical conductivity, large Seebeck coefficient and low thermal conductivity are required for high performance thermoelectric materials.

Especially, nanostructured materials are considered to be effective than bulk materials in enhancing thermoelectric figure of merit through phonon scattering at grain boundaries and interfaces. Thermoelectric materials are divided into organic and inorganic materials. For organic thermoelectric materials, many researchers have investigated by using electrically conductive polymers, such as polypyrrole, polyaniline and poly(3,4-ethylenedioxythiophene):poly(styrene sulfonate) (PEDOT/PSS)^6–10^. The conductive polymers exhibited cost effectiveness, high flexibility, low intrinsic thermal conductivity and outstanding potential for use on the human body and bent part. However, electrically conductive polymer-based thermoelectric materials are difficult to use as thermoelectric energy harvesters because of their low power factor (*S*^2^σ), high contact resistance with metal electrodes and very low thickness^11–13^. In order to achieve high output power density, the use of inorganic thermoelectric materials is inevitable. Among transition metal oxide-based inorganic thermoelectric materials, sodium cobalt oxide (NaCo_2_O_4_) is known to have high Seebeck coefficient of ~100 μV/K and *ZT* value of ~1.2 at 800 K^14–18^, in addition to non-toxicity, high thermal and chemical stability. However, the inorganic thermoelectric materials are typically expensive and brittle, which renders their application in large areas difficult.

In this study, to overcome the disadvantages of each organic and inorganic thermoelectric materials, we have fabricated a series of composite nanofiber webs composed of NaCo_2_O_4_-based ceramic nanofibers and polyacrylonitrile (PAN)-derived carbon nanofibers (CNFs), and have investigated their microstructure and thermoelectric performance by considering the relative compositions of NaCo_2_O_4_ and CNFs as well as fabrication temperature. For the purpose, NaCo_2_O_4_ precursor and PAN were dissolved in a solvent, independently, and two solutions were spun simultaneously into single nanofiber web by using dual-electrospinning technique. Then, the as-spun nanofiber webs were heat-treated at different temperatures of 600–900 °C for calcination and carbonization to develop both NaCo_2_O_4_-based nanofibers and PAN-derived carbon nanofibers (CNFs) in the final nanofiber webs. The morphology and microstructures of the as-spun and heat-treated composite nanofiber webs were characterized by using electron microscopy, energy dispersive spectroscopy, and X-ray diffraction technique. The electrical conductivity, thermal conductivity, Seebeck coefficient and thermoelectric power factor of the composite nanofiber webs were investigated by considering the compositions and microstructural features developed at different heat-treatment temperatures.

## Experimental

### Materials

Polyacrylonirile (PAN, Mw = 150,000 g/mol, Sigma Aldrich Com.) and N,N-dimethylformamide (DMF, 99.0%, Samchun Com.) were used for the preparation of CNF precursor solution. Poly(vinyl pyrrolidone) (PVP, Mw = 1,300,000 g/mol, Sigma Aldrich Com.), methanol (99.5%, Samchun Com.), sodium acetate trihydrate (99.0%, Alfa Aesar Com.) and cobalt(II) acetate tetrahydrate (98.0%, Alfa Aesar Com.) were used for preparing NaCo_2_O_4_ precursor solution for the electrospinning. All the chemicals and materials were used without further purification.

### Fabrication of composite nanofiber webs

**Figure 1:**
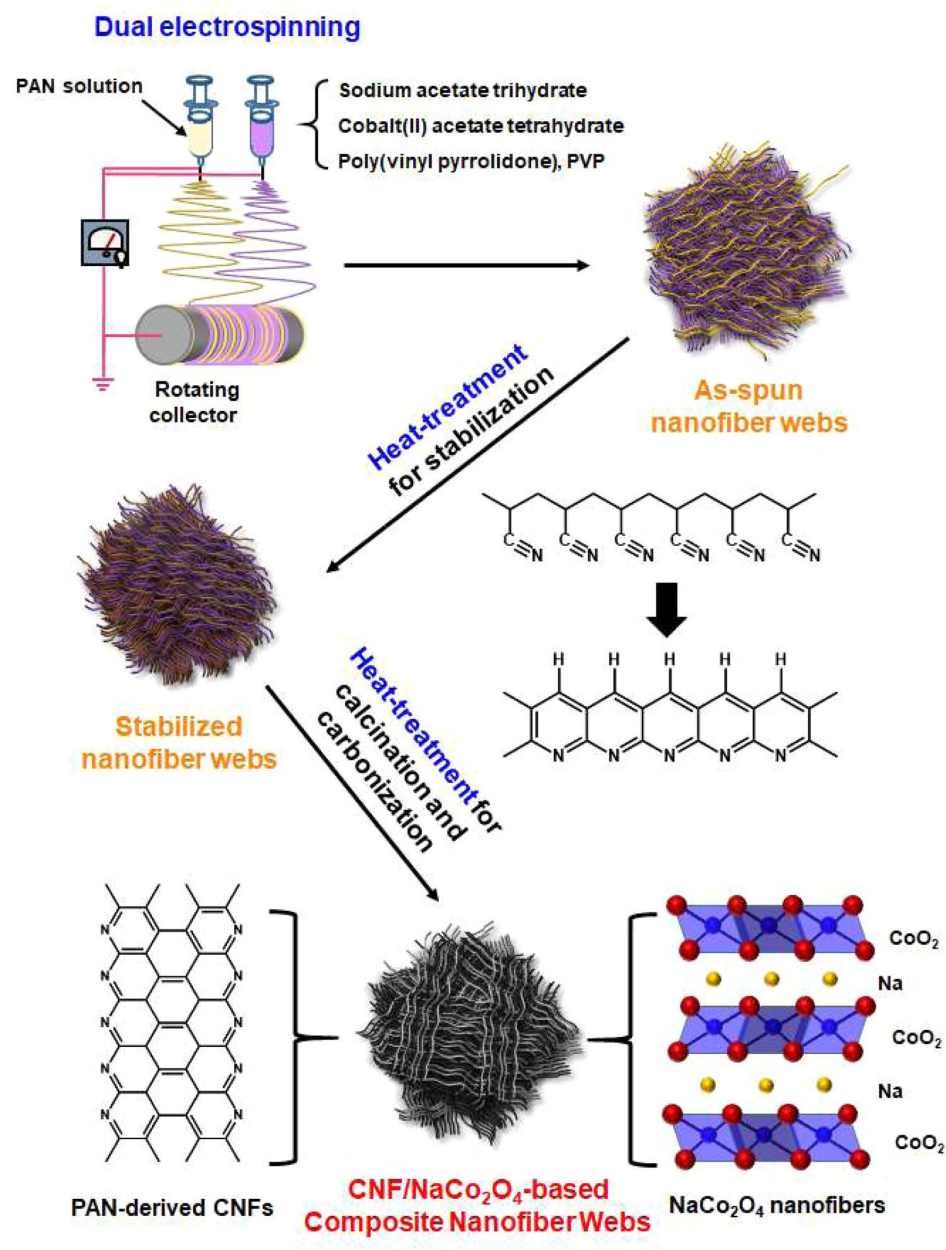
Schematic procedure for fabricating NaCo_2_O_4_/CNF-based composite nanofiber webs via dual-electrospinning and following two-step heat-treatment for stabilization and calcination/carbonization.

A series of composite nanofiber webs, which are composed of NaCo_2_O_4_ nanofibers and PAN-derived CNFs, was fabricated, as shown schematically in Fig. 1. First, three different NaCo_2_O_4_ precursor solutions for electrospinning were prepared by dissolving sodium acetate trihydrate, cobalt (II) acetate tetrahydrate, and PVP in methanol. Here, the mole ratio of sodium acetate trihydrate and cobalt (II) acetate tetrahydrate as NaCo_2_O_4_ precursors was controlled to be 1.1:2.0 and the concentrations of NaCo_2_O_4_ precursors in methanol were set to be 17.3, 32.0 and 44.0 wt%. After stirring those solutions at 20 °C for 30 min, PVP was added into each NaCo_2_O_4_ precursor solutions, which were then stirred until homogeneous solutions were obtained. A PAN solution for electrospinning was also prepared by dissolving PAN in DMF. The concentration of PAN in the solution was adjusted to be 6.0 wt%. To prepare precursor nanofiber webs via dual-electrospinning^19–21^, the PAN solution and a NaCo_2_O_4_ precursor solution, which were put in different syringes of 25 mL, were electrospun simultaneously into a rotating metal collector through a 23 gauge needle at the condition of a feeding rate of 0.5 mL/h, electric potential of 25 kV, and distance between the syringe metal tips and the collector of 30 cm. The as-spun nanofiber webs were dried at 60 °C for 2 h to remove any residual solvent and then transferred into a tubular furnace for two-step heat-treatment, which was associated with the stabilization/carbonization of PAN nanofibers as well as the calcination of NaCo_2_O_4_ precursor nanofibers. The first heat-treatment was carried out at 260 °C for 1 h under air condition for thermal stabilization of PAN nanofiber component in the dual-electrospun nanofibers. For the calcination to form NaCo_2_O_4_ ceramic nanofibers as well as for the carbonization of thermally-stabilized PAN nanofibers, the second heat-treatment was performed by heating the thermally stabilized nanofibers to different temperatures of 600–900 °C at a heating rate of 10 °C/min and holding at each final temperature for 1 h under argon gas atmosphere. The final CNF/NaCo_2_O_4_ composite nanofiber webs manufactured at different input compositions and heat-treatment temperatures were named as TMx-y, where x and y denote the content of NaCo_2_O_4_ nanofibers by wt% and the calcination/carbonization temperatures of 600–900 °C, respectively, as listed in Table 1.

**Table 1:** Input compositions and heat-treatment temperatures for fabricating CNF/NaCo_2_O_4_-based composite nanofiber webs.

Sample code	Input compositions of dual-electrospinning		Heat-treatment temperature(°C)
	PAN contentin solution (wt%)	Co acetate/Na acetate/PVPcontents in solution (wt%)	
TM0-600	6.0	0.0/0.0/6.0	600
TM0-700			700
TM0-800			800
TM0-900			900
TM15-600	6.0	13.0/4.3/6.0	600
TM15-700			700
TM15-800			800
TM15-900			900
TM30-600	6.0	24.0/8.0/6.0	600
TM30-700			700
TM30-800			800
TM30-900			900
TM45-600	6.0	32.4/11.6/6.0	600
TM45-700			700
TM45-800			800
TM45-900			900

### Characterization of composite nanofiber webs

The temperature-dependent thermal decomposition behavior of the components for fabricating CNF/NaCo_2_O_4_-based composite nanofiber webs was analyzed by using a thermogravimetric analyzer (TGA 4000, Perkin Elmer Inc.). TGA experiments were carried out under nitrogen gas condition from room temperature to 800 °C at a heating rate of 10 °C/min.

The morphological features and elemental composition of the as-spun and heat-treated composite nanofiber webs were examined by a scanning electron microscope (SEM, JSM-7000F, JEOL) equipped with an energy dispersive X-ray spectrometer (EDS). For the SEM experiment, all the nanofiber samples were coated with platinum to prevent any surface charging. The elemental composition of the composite nanofiber webs fabricated at different heat-treatment temperatures of 600–900 °C was analyzed by using EDS data.

The crystalline features of the composite nanofiber webs were identified by a high resolution X-ray diffractometer (D8 DISCOVER, Bruker AXS) with Cu-Kα radiation.

The graphitic structures of the composite nanofiber webs manufactured at different heat-treatment temperatures were characterized by using a Raman spectrometer (LabRAM HR-800).

The electrical conductivity of CNF/NaCo_2_O_4_-based composite nanofiber webs was obtained from the current-voltage (*I*-*V*) curves using an electrometer (2400, Keithley Instruments Inc.). For the electrical measurement, the nanofiber webs were cut to be 2 cm × 1 cm. The Seebeck coefficients of the freestanding composite nanofiber webs were determined using a home-built setup consisting of a temperature control part, thermoelectric measurement part, and measurement stage. Silver electrodes isolated by a distance of 2 cm were formed on the composite nanofiber webs for the ohmic contact between the surface of the nanofiber webs and the measurement probe. The temperature difference between the ends of the nanofiber webs was controlled elaborately by using Peltier devices, and the Seebeck voltage was measured as a function of the temperature difference (Δ*T* = 4–8 °C) from room temperature of 25 °C. The Peltier devices for hot and cold parts were controlled by a Keithley 2200 power source and a Keithley 2460 source meter. The electrical conductivity and Seebeck coefficient were used for calculating the thermoelectric power factor of the composite nanofiber webs.

The thermal conductivity of the composite nanofiber webs was characterized by measuring the density, thermal diffusivity, and specific heat capacity. The density was evaluated by measuring the weight and volume of the composite nanofiber webs with 1 cm × 1 cm. The thermal diffusivity and specific heat capacity were measured by using a laser flash apparatus (LFA 467, NETZSCH Inc.) and a differential scanning calorimeter (DSC 6000, Perkin Elmer Inc.), respectively. For each sample, five different measurements were carried out for reproducibility.

## Results and Discussion

### Thermal decomposition behavior of nanofiber components

**Figure 2:**
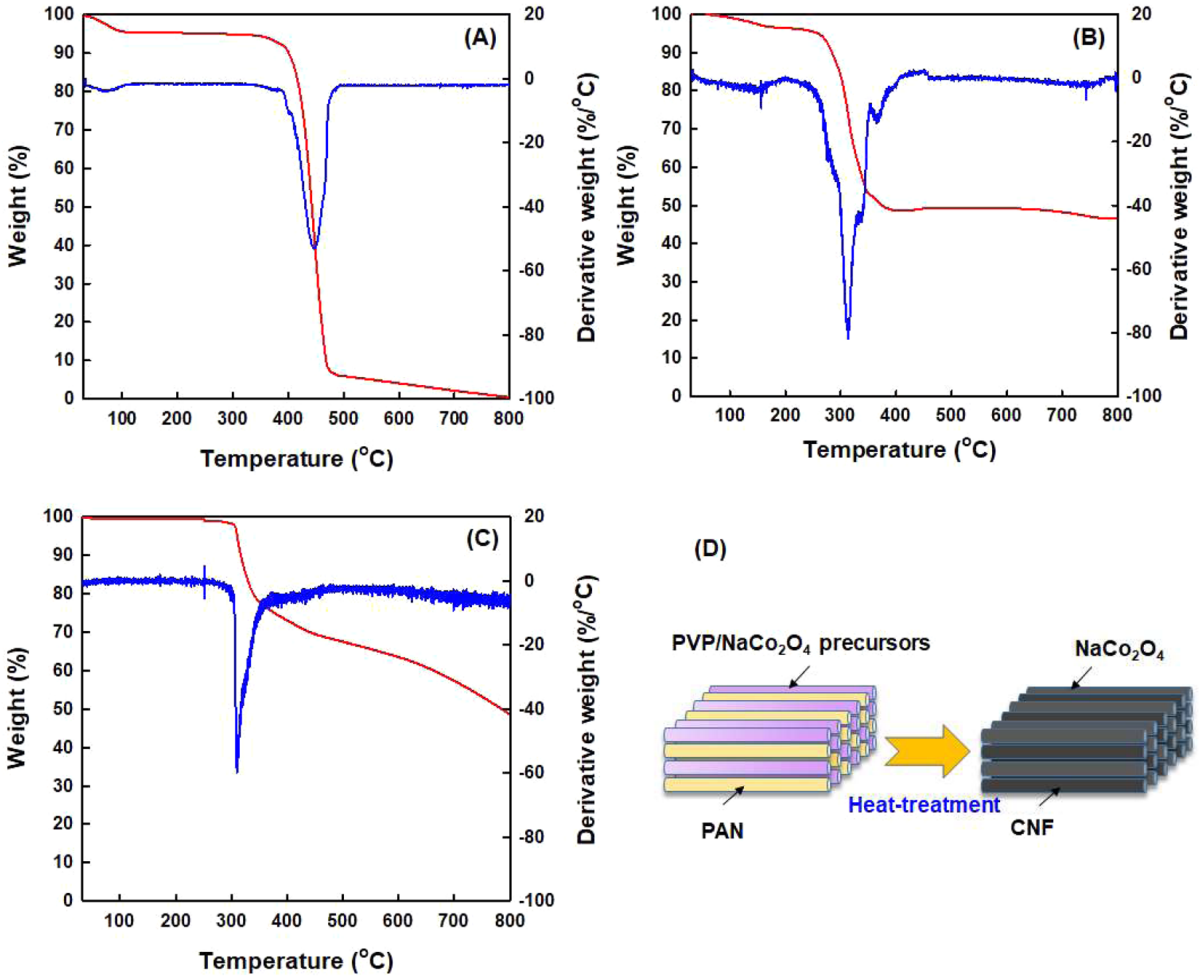
TGA and DTG curves of (**A**) PVP, (**B**) NaCo_2_O_4_ precursor, and (**C**) stabilized PAN. (**D**) Heat-treatment for calcination and carbonization.

**Figure 3:**
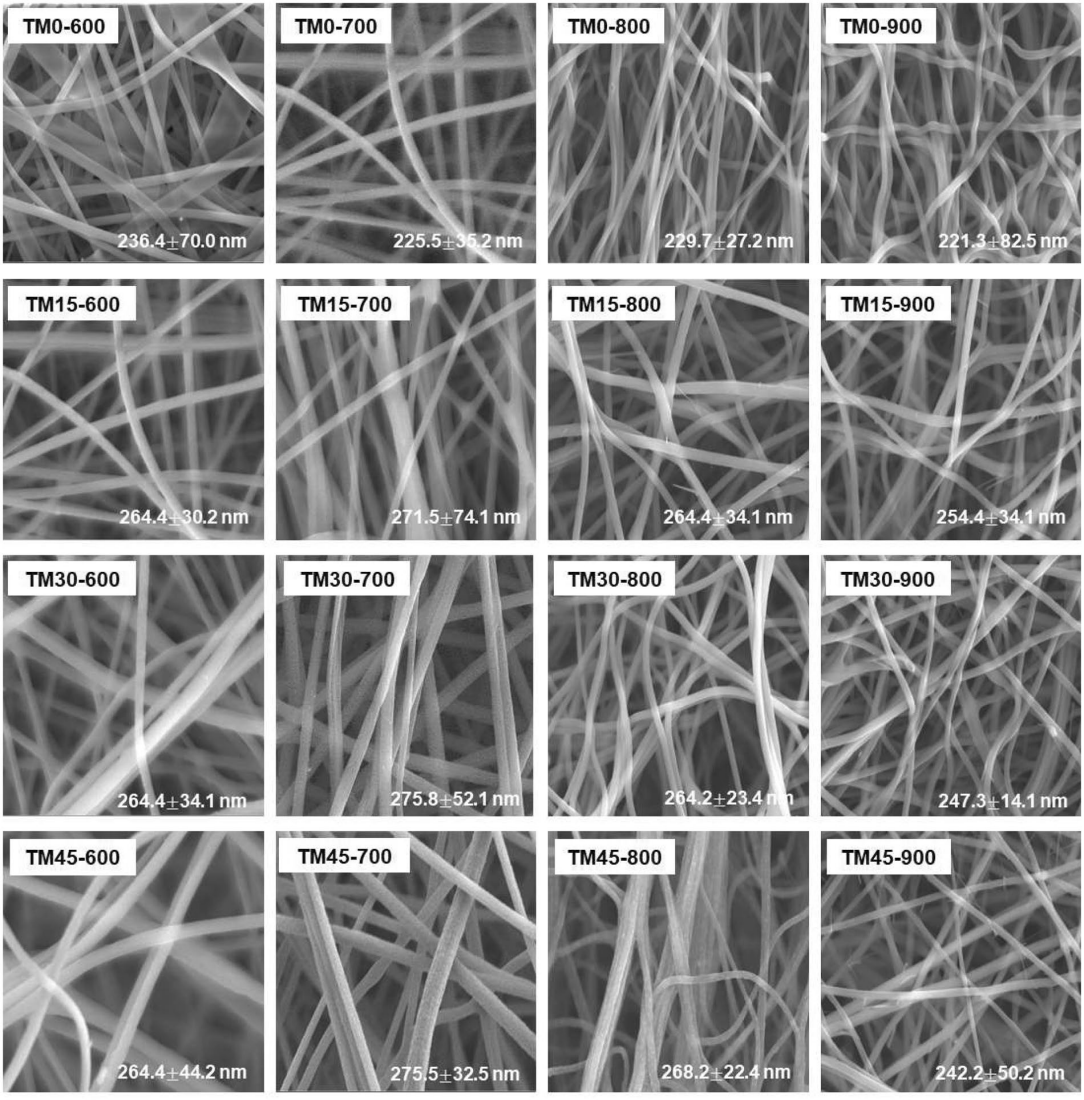
SEM images of CNF/NaCo_2_O_4_-based composite nanofiber webs fabricated at different input compositions and heat-treatment temperatures of 600-900 °C.

To identify the thermal decomposition behavior of the components used for fabricating CNF/NaCo_2_O_4_-based composite nanofibers, the TGA and DTG curves of neat PVP nanofiber, stabilized PAN nanofibers and NaCo_2_O_4_ precursor powders were obtained, as shown in Fig. 2. For the neat PVP nanofibers, a dominant thermal decomposition took place at ~460 °C and the residue at 800 °C was ~0% (Fig. 2A). In case of NaCo_2_O_4_ precursor powders, a main thermal decomposition was detected at the temperature range of 250–350 °C and the residue at 800 °C was above 40% (Fig. 2B). For the thermally-stabilized PAN nanofibers, single thermal decomposition occurs dominantly at ~300 °C by remaining relatively high residue of ~50 wt% at 800 °C (Fig. 2C). It is thus valid to contend that, during the heat-treatments for the simultaneous calcination/carbonization process of the as-spun nanofiber webs, the PVP component in PVP/NaCo_2_O_4_ precursor-based nanofibers was decomposed completely above 460 °C by forming NaCo_2_O_4_ ceramic nanofibers and the thermally-stabilized PAN nanofibers can be developed into CNFs, as represented schematically in Fig. 2D and 3.

### Morphology and composition analyses of composite nanofiber webs

Fig. 3 shows the SEM images of as-spun and heat-treated composite nanofiber webs, which were fabricated at different input compositions and heat-treatment temperatures of 600–900 °C. The SEM images showed that all the nanofiber webs are composed of cylindrical nanofibers. The average diameters of all the nanofibers were measured to be in the range of 200–300 nm, although they decrease slightly with increasing the heat-treatment temperature for the calcination/carbonization.

**Figure 4:**
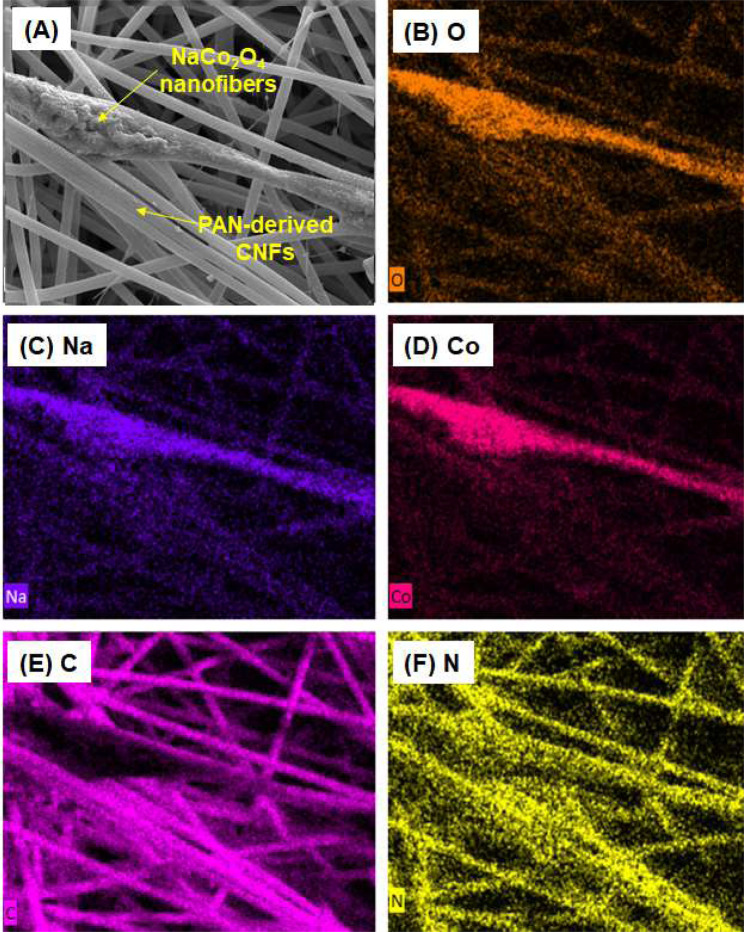
(**A**) SEM and (**B**–**F**) EDS elemental mapping images of TM45-70 composite nanofiber web.

To identify the presence of PAN-derived CNFs and NaCo_2_O_4_-based nanofibers in the composite nanofiber webs, typical SEM and EDS mapping images of TM45–700 were obtained, as can be seen in Fig. 4. The elemental mapping images support the fact that both CNFs and NaCo_2_O_4_ nanofibers coexist in the composite nanofiber webs owing to the dual-electrospinning process.

The relative elemental compositions of CNF/NaCo_2_O_4_-based composite nanofiber webs manufactured at different input compositions and heat-treatment temperatures of 600–900 °C were determined by using EDS data, as listed in Table 2. It was found that the relative composition of NaCo_2_O_4_ nanofibers in the composite nanofibers fabricated at the heat-treatment of 600 and 700 °C increased with the increment of the input composition of NaCo_2_O_4_ precursors during the dual-electrospinning. However, for the composite nanofiber webs manufactured at relatively high heat-treatment temperatures of 800 and 900 °C, the relative contents of sodium, oxygen and cobalt were found to be lowered owing to the thermal decomposition of NaCo_2_O_4_ precursors during the heat-treatment process. It is thus speculated that the thermoelectric performance would be reduced for the composite nanofiber webs manufactured at high heat-treatment temperatures of 800–900 °C, which results from relatively low composition of thermoelectric NaCo_2_O_4_ nanofibers, in comparison to CNFs.

**Table 2:** Relative elemental compositions of CNF/NaCo_2_O_4_-based composite nanofiber webs fabricated at different input compositions and heat-treatment temperatures of 600-900 °C.

Sample code	Elemental composition (wt%)				
	C	N	O	Na	Co
TM0-700	86.50	11.32	2.18	—	—
TM15-600	52.11	7.23	10.69	9.02	20.95
TM15-700	44.78	10.45	13.63	9.71	21.42
TM15-800	69.85	13.95	4.95	1.84	9.41
TM15-900	75.55	15.21	5.75	1.85	2.64
TM30-600	49.88	8.18	12.15	9.25	20.54
TM30-700	49.05	9.03	12.00	6.82	23.11
TM30-800	70.35	10.96	2.25	1.97	14.47
TM30-900	85.71	5.81	5.17	1.02	2.28
TM45-600	50.24	7.58	11.15	10.12	20.91
TM45-700	49.24	11.81	9.02	9.82	20.11
TM45-800	67.90	9.78	8.73	3.16	10.44
TM45-900	70.67	15.11	7.02	3.66	3.54

### Structural characterization of composite nanofiber webs

**Figure 5:**
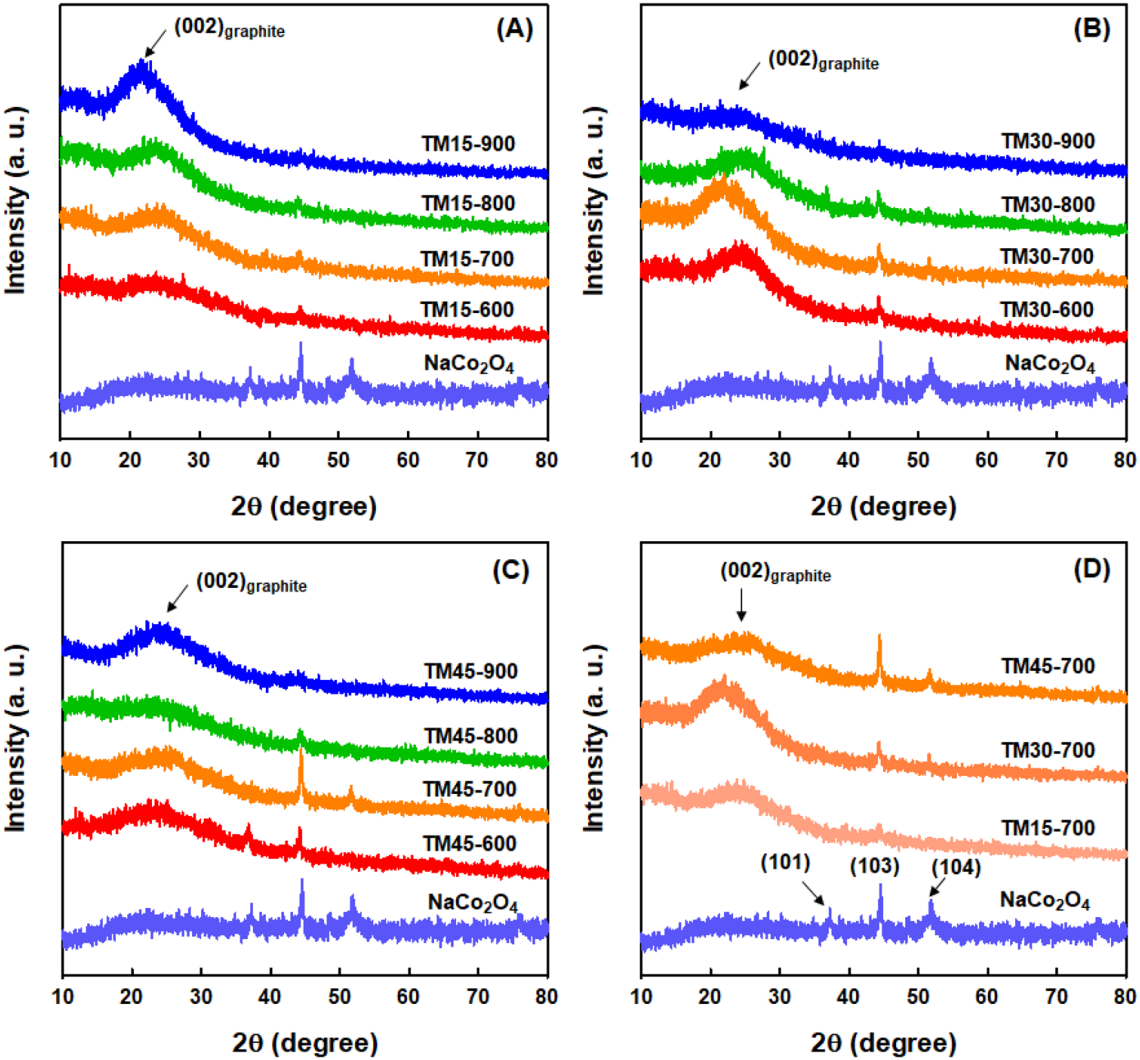
X-ray diffraction patterns of NaCo_2_O_4_ powder and CNF/NaCo_2_O_4_-based composite nanofiber webs fabricated at different input compositions and heat-treatment temperatures of 600-900 °C.

The crystalline features of CNF/NaCo_2_O_4_-based composite nanofiber webs fabricated at different input compositions and heat-treatment temperatures of 600–900 °C were examined by X-ray diffraction method, as shown in Fig. 5. NaCo_2_O_4_ powders prepared by sol-gel method exhibited sharp diffraction peaks, which are associated with (101), (103) and (104) planes of *γ*-phase NaCo_2_O_4_ crystals^22^. In addition, all CNF/NaCo_2_O_4_-based composite nanofiber webs exhibited a broad diffraction peak at ~23°, which is related to the reflection of (002) plane of graphitic structure of CNFs. On the other hand, the composite nanofiber webs fabricated at 600–700 °C exhibited distinctive diffraction peaks associated with (101), (103) and (104) planes of *γ*-phase NaCo_2_O_4_ crystals, while the composite nanofiber webs manufactured at 800–900 °C showed relatively weak diffraction peaks related with NaCo_2_O_4_ crystals, as can be seen in Fig. 5A–C. As a result, most high crystalline feature of *γ*-phase NaCo_2_O_4_ crystals was observed for the composite nanofiber webs heat-treated at 700 °C. In addition, the diffraction peaks of *γ*-phase NaCo_2_O_4_ crystals were found to increase with the input composition of NaCo_2_O_4_ precursors during the dual-electrospinning process, as can be seen in Fig. 5D. These results are quite consistent with the elemental analyses of above EDS data in Table 2. It was thus expected that the thermoelectric performance of the composite nanofiber webs fabricated at 800–900 °C would be reduced owing to the lowered contents of NaCo_2_O_4_ nanofibers in the composite nanofiber webs.

**Figure 6:**
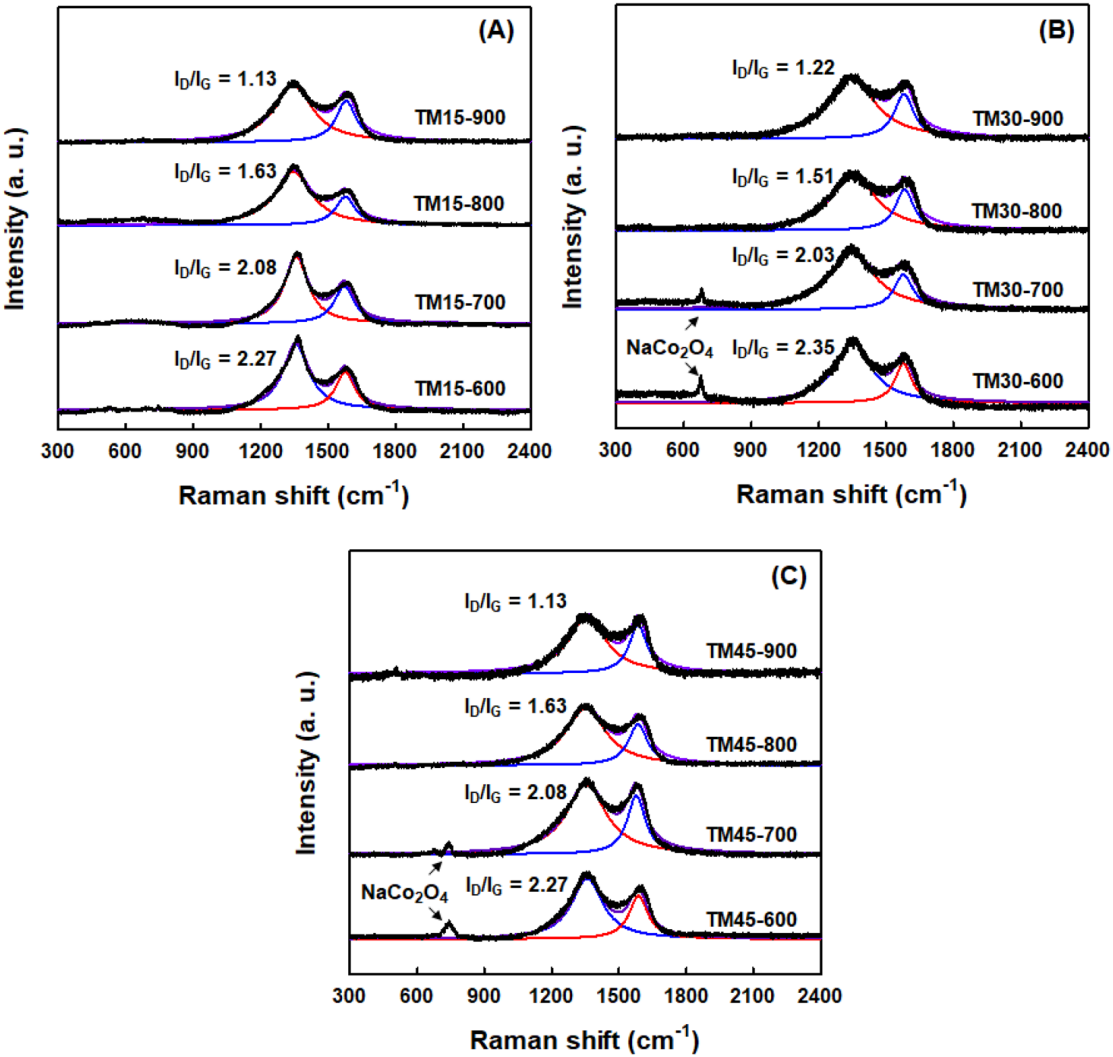
Raman spectra of CNF/NaCo_2_O_4_-based composite nanofiber webs fabricated at different input compositions and heat-treatment temperatures of 600-900 °C.

The Raman spectra of CNF/NaCo_2_O_4_-based composite nanofiber webs fabricated at different input compositions and heat-treatment temperatures of 600–900 °C were represented in Fig. 6. All the composite nanofiber webs showed two strong scattering bands at 1350 cm^−1^ and 1580 cm^−1^, which correspond to *D* band and *G* band, respectively. It is known that the *G* band stems from an ordered graphitic carbon structure with *sp*^2^ hybridization, whereas the *D* band originates from carbon defects with *sp*^3^ hybridization. When the integrated intensity ratio of the *D* and *G* bands (*I*_D_/*I*_G_) was compared for all composite nanofiber webs, it was found to decrease with the increment of the heat-treatment temperatures for calcination/carbonization as well as the input compositions of NaCo_2_O_4_ precursors. This result supports the fact that more ordered graphitic structure was developed for the composite nanofiber webs manufactured at higher heat-treatment temperature. On the other hand, a typical Raman band of NaCo_2_O_4_ was also detected at 680 cm^−1^, which was dominant for the composite nanofiber webs heat-treated at 600 and 700 °C^23–25^. The relative Raman intensity data of NaCo_2_O_4_ are found to be also consistent with the data from EDS-based elemental analyses and X-ray diffraction data.

### Thermoelectric properties of composite nanofiber webs

**Figure 7:**
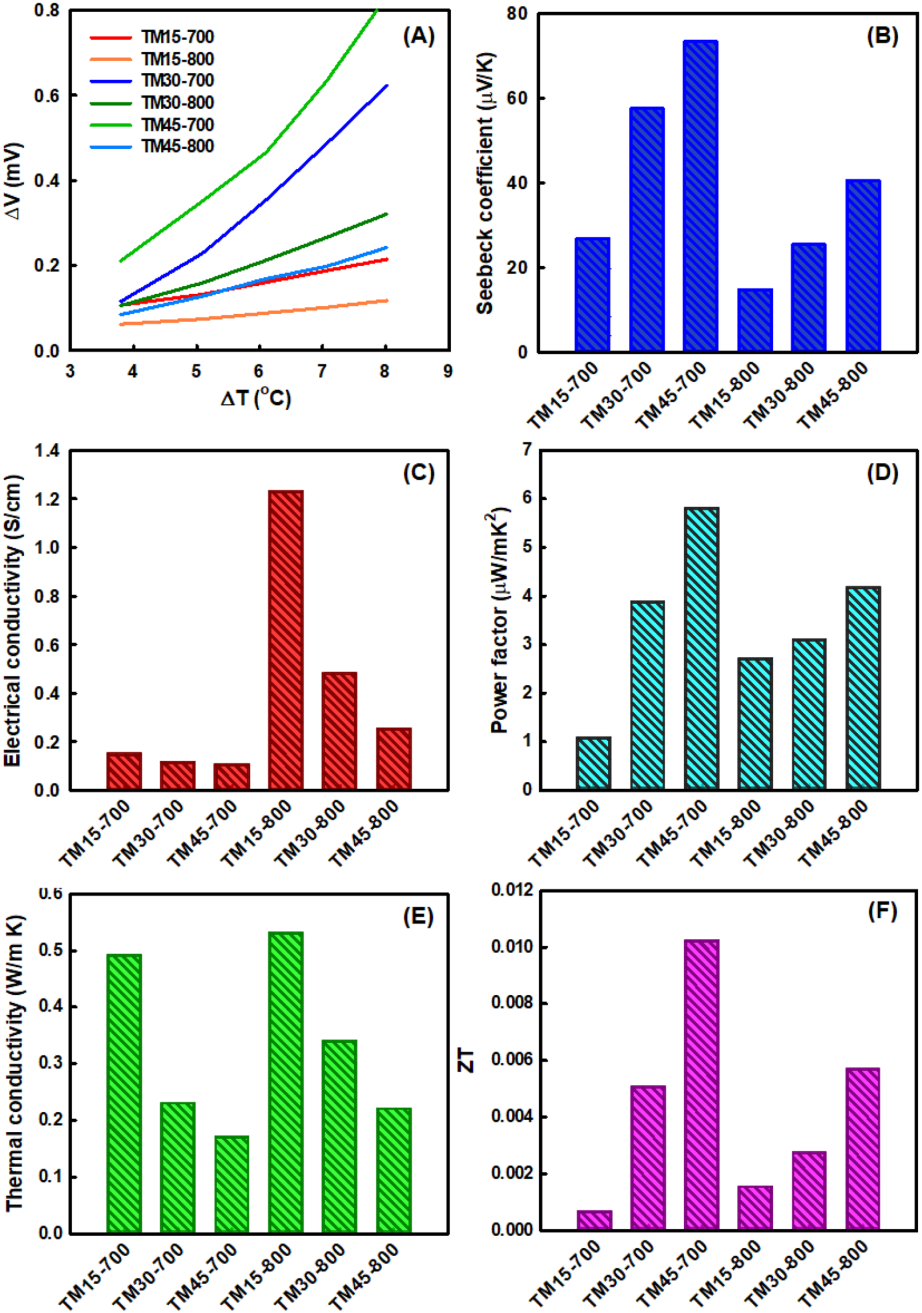
(**A**) Open circuit voltage *vs*. temperature difference plots, (**B**) Seebeck coefficient, (**C**) electrical conductivity, (**D**) power factor, (**E**) thermal conductivity, and (**E**) figure of merit (*ZT*) of CNF/NaCo_2_O_4_-based composite nanofiber webs fabricated at different input compositions and heat-treatment temperatures of 700 and 800 °C.

To evaluate the total thermoelectric output voltage of CNF/NaCo_2_O_4_-based composite nanofiber webs, the open circuit voltage (Δ*V*) was obtained as a function of temperature difference (Δ*T*), as shown in Fig. 7A. The increment of open circuit voltages with the temperature difference was found to be higher for CNF/NaCo_2_O_4_-based composite nanofibers manufactured at higher input composition of NaCo_2_O_4_ precursor as well as heat-treatment temperatures. From the slopes of the open circuit voltage *versus* temperature difference plots in Fig. 7A, the Seebeck coefficient, *S*, can be determined by using the following equation:

1 $$S=\frac{\Delta V}{\Delta T}$$

The Seebeck coefficients evaluated for the composite nanofiber webs manufactured at different input compositions of NaCo_2_O_4_ precursors and heat-treatment temperatures of 700 and 800 °C were in the range of 14.83–73.28 μV/K at low temperature differences of 4–8 °C, as represented in Fig. 7B. At a same input composition of NaCo_2_O_4_ precursors, the Seebeck coefficients of the composite nanofiber webs manufactured at 700 °C were higher than those of the nanofiber webs fabricated at 800 °C. In addition, at a same heat-treat temperature, the Seebeck coefficients were higher for the composite nanofiber webs fabricated with higher input compositions of NaCo_2_O_4_ precursors. As a result, TM45-700 sample was found to have the highest Seebeck coefficient of ~73 μV/K.

The electrical property of CNF/NaCo_2_O_4_-based composite nanofibers was also characterized by evaluating the electrical conductivity (σ, S/cm) based on following equation:

2 $$\sigma =\frac{1}{R}\frac{A}{L}$$

where *R* is the electrical resistance, *L* is the sample length between electrodes, and *A* is the cross-section area of sample. Figure 7C shows that the electrical conductivity of the composite nanofiber webs increased with increasing the heat-treatment temperature for the calcination and carbonization as well as with decreasing the input composition of PAN component as conductive CNFs. Accordingly, TM15-800 sample was determined to have the highest electrical conductivity of 1.23 S/cm.

From the Seebeck coefficient and electrical conductivity data of Fig. 7B,C, it is valid to contend that the Seebeck coefficient increased and the electrical conductivity decreased, as the heat-treatment temperature increased and the input composition of PAN decreased. It is conjectured that the high Seebeck coefficient and low electrical conductivity of TM45-700 is associated with the low carrier concentration, because the Seebeck coefficient is inversely proportional to the carrier concentration, unlike the electrical conductivity. Furthermore, the charge carriers are likely to be affected by the boundaries thermoelectric NaCo_2_O_4_ crystals in the nanofiber webs, thus reducing the electrical conductivity.

The power factor, *S*^2^σ, is often claimed as the performance parameter of thermoelectric materials. From the Seebeck coefficient and electrical conductivity data of Fig. 7B,C, the power factor values of CNF/NaCo_2_O_4_-based composite nanofiber webs were evaluated to be in the range of 1.07–5.79 μW/mK^2^, as shown in Fig. 7D. Among the composite nanofiber webs, TM45-700 sample was found to have the highest power factor value of 5.79 μW/mK^2^, which is owing to the dominant contribution of the Seebeck coefficient, compared to the electrical conductivity. In order to compare the thermoelectric performance of CNF/NaCo_2_O_4_ composite nanofibers with the thermoelectric nanofibers, films and nanowires reported in literatures, the Seebeck coefficient, electrical conductivity and power factor values are summarized in Table 3. It is noteworthy that the Seebeck coefficient and electrical conductivity of CNF/NaCo_2_O_4_-based composite nanofibers are far higher and even lower than those of other thermoelectric nanofibers in literatures, respectively^5,26–33^. Nonetheless, the power factor value of CNF/NaCo_2_O_4_ composite nanofiber webs was found to be higher than or comparable to other thermoelectric nanofibers.

**Table 3:** Structural features and physical properties of representative thermoelectric materials.

Sample	Shape	Preparation Method	Seebeck coefficient(μV/K)	Electrical conductivity(S/cm)	Power factor (μW/mK^2^)	Reference
CNF/NaCo_2_O_4_	Nanofiber	Electrospinning	73.82	0.10	5.79	This study
PEDOT:PSS/PVA@Ag	Nanofiber	Electrospinning	20	40	1.2	Jin *et al*.^5^
PP/CNF	Composite	Compression-molding	8.5	—	1.75 × 10^−3^	Paleo *et al*.^26^
PAN/Bi_2_Te_3_	Nanofiber	Electrospinning	4	—	—	Gan *et al*.^27^
PAN/lignin-derived CNFs	Nanofiber	Electrospinning	10	8.2	9.27	Dalton *et al*.^28^
PEDOT	Nanofiber	Reverse microemulsion polymerization	48	71.4	16.4	Hu *et al*.^29^
P3HT	Nanofiber	Whisker method	48.8	12.6	3.7	Hiura *et al*.^30^
PEDOT/CNT	Composite film	*In situ* polymerization	12	15	0.2294	Zhang *et al*.^31^
PEDOT/CNT	Hybrid film	Spray- and spin-coating	~1100	~7	~1000	Wang *et al*.^32^
PANI/Bi_2_Te_3_	Composite	*In situ* polymerization	35	12	1.57	Chatterjee *et al*.^33^

The thermal conductivity, *κ*, of CNF/NaCo_2_O_4_-based composite nanofiber webs can be determined by using following equation:

3 $$\kappa =\alpha \,\rho \,{C}_{{\rm{p}}}$$

where *α* is the thermal diffusivity, *ρ* is the density, and *C*_p_ is specific heat capacity. The experimentally measured *α*, *ρ* and *C*_p_ values were summarized in Table 4 and the calculated thermal conductivity data are represented in Fig. 7E. The thermal conductivity of the composite nanofiber webs was evaluated to be in the range of 0.17–0.53 W/mK, which decreases with the increasing the input composition of NaCo_2_O_4_ and the heat-treatment temperature. Overall, based on the power factor and thermal conductivity data, the figure of merit, *ZT*, of CNF/NaCo_2_O_4_ composite nanofiber webs at 30 °C could be calculated, as shown in Fig. 7F. It was found that a maximum *ZT* value of ~0.01 was attained for TM45-700 sample with a high power factor of 5.79 μW/mK^2^ and a low thermal conductivity of 0.17 W/mK. It is thus believed that the composite nanofibers webs, which consist of electrically conductive CNF nanofibers and thermoelectric NaCo_2_O_4_ nanofibers, can be utilized as potential thermoelectric materials in aspects of facile fabrication, mass production, and applicability to wearable energy harvesting devices.

**Table 4:** Density, thermal diffusivity, and specific heat capacity of CNF/NaCo_2_O_4_-based composite nanofiber webs.

Sample code	Thermal diffusivity (mm^2^/s)	Density (mg/mm^3^)	Specific heat capacity (J/g K)
TM15-700	0.534	0.046	0.050
TM15-800	0.588	0.045	0.049
TM30-700	0.424	0.049	0.090
TM30-800	0.529	0.051	0.079
TM45-700	0.358	0.059	0.124
TM45-800	0.417	0.053	0.100

## Conclusion

In summary, a series of thermoelectric CNF/NaCo_2_O_4_-based composite nanofiber webs was fabricated by using dual-electrospinning and following heat-treatment at different temperatures of 500–900 °C for simultaneous carbonization and calcination. The SEM and EDS data revealed that both crystalline NaCo_2_O_4_ nanofibers and CNFs were successfully developed in the composite nanofiber webs and that the relative contents of NaCo_2_O_4_ nanofibers decreased rapidly for the composite nanofibers fabricated at high heat-treatment temperatures of 800–900 °C. Consistently, the X-ray diffraction results showed that the diffraction peaks of γ-form NaCo_2_O_4_ crystals decreased rapidly for the composite nanofiber webs heat-treated at 800–900 °C, while all the composite nanofiber webs showed a broad peak at ~22°, which corresponds to (002) reflection of CNFs with partially ordered graphitic structure. For CNF/NaCo_2_O_4_-based composite nanofiber webs heat-treated at 700 and 800 °C, the Seebeck coefficient increased and the electrical conductivity decreased, as the input composition of PAN decreased and the heat-treatment temperature increased. Owing to the trade-off effects of the input composition and heat-treatment temperature on the Seebeck coefficient, electric conductivity and thermal conductivity of the composite nanofiber webs, TM45-700 sample, which was fabricated with 45 wt% input composition of NaCo_2_O_4_ precursor and heat-treatment of 700 °C_,_ was characterized to have a maximum power factor of ~5.79 μW/mK^2^ and a figure of merit of ~0.01 at quite low temperature differences of 4–8 °C. Accordingly, it is valid to contend that CNF/NaCo_2_O_4_-based composite nanofiber webs, which are fabricated by facile and efficient dual-electrospinning and heat-treatment, can be applied as a promising thermoelectric material for wearable devices harvesting electricity from waste heat of industry and human body.
